# Does extremely early expression of colostrum after very preterm birth improve mother’s own milk quantity? A cohort study

**DOI:** 10.1136/archdischild-2023-326784

**Published:** 2024-03-04

**Authors:** Ilana Levene, Maria A Quigley, Mary Fewtrell, Frances O’Brien

**Affiliations:** 1 National Perinatal Epidemiology Unit, Nuffield Department of Population Health, Oxford University, Oxford, UK; 2 UCL Great Ormond Street Institute of Child Health, University College London, London, UK; 3 Neonatal Unit, John Radcliffe Hospital, Oxford, Oxfordshire, UK

**Keywords:** Neonatology, Intensive Care Units, Neonatal

## Abstract

**Objective:**

Assess the relationship of time to first expression after very preterm birth and mothers’ own milk quantity.

**Design:**

A cohort study (nested within a randomised trial).

**Setting:**

Four neonatal units in the UK.

**Patients:**

132 mothers of single or twin infants born at 23+0 to 31+6 weeks postmenstrual age.

**Exposures:**

Time to the first attempt to express after birth.

**Primary outcomes:**

24-hour mother’s own milk yield on days 4, 14 and 21 after birth.

**Results:**

Median time to first expression attempt was 6 hours. 51.7% expressed within 6 hours of birth (62/120) and 48.3% expressed more than 6 hours after birth (58/120). Expressing within 6 hours of birth was associated with higher milk yield on day 4 (88.3 g, 95% CI 7.1 to 169.4) and day 14 (155.7 g, 95% CI 12.2 to 299.3) but not on day 21 (73.6 g, 95% CI −91.4 to 238.7). There was an interaction between expressing frequency and time to first expression (p<0.005), with increased expressing frequency being associated with higher yield only in those who expressed within 6 hours. Expressing within 2 hours of birth was not associated with further milk yield increase.

**Conclusions:**

Mothers who expressed within 6 hours of birth had higher milk yield, and a greater yield per expressing session, in the first 3 weeks after birth. This information will be highly motivating for families and the clinicians supporting them. There was no evidence of further benefit of extremely early expression (first 2 hours after birth).

**Trial registration number:**

ISRCTN 16356650.

**WHAT IS ALREADY KNOWN ON THIS TOPIC**

- Increased quantity of mother’s own milk provided to very preterm infants is associated with lower morbidity and mortality.
- Initiating expression of colostrum sooner after birth is associated with improved lactation outcomes in comparison to later initiation, when direct breast feeding is not possible.
- International recommendations range from expressing within 2 hours of birth to within 6 hours of birth. Many clinical teams are working on quality improvement projects in this area.

**WHAT THIS STUDY ADDS**

- Expressing within 6 hours of very preterm birth was associated with a significant increase in mother’s milk yield during the first weeks after birth.
- Mothers who expressed within 6 hours of very preterm birth had greater milk yield per expressing session, particularly on day 4 after birth.
- Expressing within 2 hours of birth did not give a significant further advantage.

**HOW THIS STUDY MIGHT AFFECT RESEARCH, PRACTICE OR POLICY**

- These findings are useful to clinical teams working in quality improvement to set evidence-based targets for the time to first expression when direct breast feeding is not possible.
- These findings are useful to motivate staff and parents on the value of early initiation of expression.

## Introduction

Breast feeding is the biological norm and mother’s own milk (MOM) has a particular impact on preterm infants, with respect to neurodevelopmental outcomes,^1^ necrotising enterocolitis^2^ and retinopathy of prematurity.^3^ Increasing exclusive human milk for preterm infants is a WHO research priority.^4^ There are also important impacts on maternal health (such as breast cancer).^5^

In the UK, mothers whose infants are not able to directly breast feed are recommended (by the UNICEF UK Baby Friendly Initiative) to start expressing colostrum within 2 hours of birth.^6^ International guidance recommends expression within 1–3 hours^7^ or within 6 hours of birth.^8^ This can be challenging due to the trauma of very preterm birth, which is often associated with maternal complications requiring urgent treatment, such as pre-eclampsia and haemorrhage. It can also be challenging because of structural barriers, such as the division of postnatal responsibilities between maternity and neonatal ‘silos’ and because of competition with other ‘golden hour’ activities for maternity and neonatal teams.^9^ Due to these significant barriers to achieving a target of very early expression after very preterm birth, it is important that recommendations have a clear evidence base.

Typical lactation physiology at term is based on an infant suckling at the breast after birth. Early first suckling experience is linked to increased milk production in the days after birth.^10^ Suckling in the first 2 hours after birth was associated with an adjusted increase in MOM at day 4 after birth of 98 mL in primiparous women and 124 mL in multiparous women.^10^ Initiating breast feeding more than an hour after birth was associated with an adjusted OR of 1.6 for early breastfeeding cessation.^11^ An interventional study comparing first breast feed within 10 min of birth to first breast feed at 4–6 hours after birth showed that the early group had a longer breastfeeding duration.^12^

Two randomised trials on time to first expression after the birth of a very preterm, very low birth weight infant have taken place, with conflicting results.^13 14^ In the first pilot study with 20 participants, there was a mean increase in expressed MOM of 346 mL per day, 3 weeks after birth, in the group allocated to expression within an hour of birth, compared with those expressing between 1 and 6 hours after birth. However, the fully powered trial by the same researchers did not replicate these results, showing no increase in long-term milk yield when expressing less than an hour or 1–3 hours from birth, in comparison to between 3 and 6 hours from birth. The authors concluded that expressing within 3–6 hours of birth is an acceptable goal, with no further benefit of earlier initiation.

Although earlier time to first expression is generally associated with improved lactation outcomes in observational studies, including many that specifically examine a 6-hour cut-off,^15–20^ very few have examined shorter times within the first 6 hours.^17^ The evidence for current clinical recommendations to express at very early time points such as less than 1–3 hours after birth is therefore weak, whereas advice to express within 6 hours of birth is stronger.

A final piece of notable evidence is a study reporting an interaction between time to first expression and expressing frequency. Participants initiating expression early and expressing infrequently had much higher milk yield than those who initiated expression late and expressed infrequently, a differential relationship that was not seen when participants expressed frequently.^21^ It is important to attempt to replicate this finding.

The objectives of this study were therefore to study the relationship of time to the first attempt to express colostrum with MOM quantity, with particular reference to clinical guidance thresholds.

## Methods

### Trial design

This report uses data collected for a randomised controlled trial of a relaxation intervention. A detailed protocol has been published.^22^ In brief, 132 people who had given birth to one or two infants between 23+0 and 31+6 weeks’ postmenstrual age (PMA) were recruited in four tertiary or local neonatal units in England. The trial was powered in relation to the randomised intervention, for a primary outcome of the highest 24-hour milk yield recorded on days 4, 14 or 21 after birth, as described in the protocol.^22^

Three of the four units had UNICEF UK Baby Friendly Initiative level three accreditation and all had free provision of hospital-grade breast pumps both at the hospital and at home for at least the first 3 weeks after birth. All units train staff to recommend first expression within 2 hours of birth, expressing frequency of 8–10 times a day on an ongoing basis, and the use of electric double pumping by day 4 after birth. All units have donor human milk available for high-risk infants in the early period, but would only offer donor human milk at 36 weeks’ PMA in exceptional circumstances.

Participants were given a portable scale accurate to 0.1 g (Kabalo) and filled in logs each time they expressed milk for 24 hours on three specific time points (days 4, 14 and 21 after birth). The specific gravity of human milk is 1.03 so 1 g and 1 mL are considered near equivalent.^23^ Baseline data, including time to first expression, were provided by the participant at recruitment, which could take place between birth and day 3 after birth. Infant feeding status was assessed by text message at 36 weeks’ PMA, or via infant medical notes if there was no response.

This exploratory analysis was prespecified.

### Statistical analysis

Analysis of expressed milk yield used linear regression. The planned analysis was to use multilevel modelling, including data measured on days 4, 14 and 21 in a single primary outcome analysis. However, there were multiple statistical interactions of time with key explanatory variables so instead linear regression was performed separately at each time point. Secondary outcome analysis of exclusive MOM at 36 weeks’ PMA, and of the timing of first expression after birth, used Poisson regression with robust SE. The analysis took place on a complete case basis.

Potential adjustment variables were sociodemographic and perinatal factors, and dynamic expressing related factors such as the type of expression used and skin-to-skin contact duration. These potential confounders were identified from the literature. Variables were included as candidates for multivariable analysis if they showed association with the outcome in univariable analysis with p<0.2 and were retained in the final model if p<0.05 after stepwise removal. The randomised allocation variable was retained in the model regardless of the significance level. The prespecified hypothesis of an interaction between expressing frequency and time to first expression was assessed with a test for statistical interaction.

The index of multiple deprivation quintile was derived from participant postcode—this measure is assigned by the UK government according to multiple measures of deprivation.

## Results

### Cohort description

The baseline characteristics of the cohort are presented in table 1. Mean maternal age was 32.8±6.3 years and gestation at birth was 27.8±2.4 weeks’ PMA. Overall, 70.2% planned to exclusively MOM feed, 59.7% were primiparous, 17.5% were black, 18.3% were Asian, 38.2% lived in the most deprived two quintiles of England and 31.7% left full-time education before 19 years of age.

**Table 1:** Baseline characteristics of the cohort

	Whole cohort (n=132)
**Ethnic group; n (%**)	
Asian or Asian British	22 (18.3)
Black, African, Black British or Caribbean	21 (17.5)
Other	5 (4.2)
White	72 (60.0)
Prefer not to say/missing	12
**Maternal age at randomisation in years; mean (SD**)	32.8 (6.3)
**Index of Multiple Deprivation Quintile; n (%**)	
1 (most deprived)	24 (18.3)
2	26 (19.8)
3	22 (16.8)
4	31 (23.7)
5 (least deprived)	28 (21.4)
Missing	1
**Age when left full-time education; n (%**)	
16 years old or less	14 (11.7)
17 or 18 years old	24 (20.0)
19 years old or more	82 (68.3)
Prefer not to say/missing	12
**Lives with a partner; n (%**)	108 (89.3)
Missing	11
**Smoking at birth; n (%**)	8 (6.6)
Missing	11
**Baseline intention to exclusively human milk feed at discharge; n (%**)	85 (70.2)
Missing	11
**Mode of birth; n (%**)	
Vaginal	57 (43.2)
Caesarean	75 (56.8)
**Multiple pregnancy; n (%**)	20 (15.2)
**Primiparous; n (%**)	74 (59.7)
Missing	8
**Multipara only, N**	50
**Previous human milk feeding experience; n (%**)	
None	5 (10.2)
<6 months	18 (36.7)
≥6 months	26 (53.1)
Missing	1
**Infant born in a recruiting centre (at least one if twins); n (%**)	123 (93.2)
**Infant gestational age at birth; n (%**)	
23 to <26 weeks	34 (25.8)
26 to <28 weeks	37 (28.0)
28 to <30 weeks	29 (22.0)
30 to <32 weeks	32 (24.2)
**Infant’s Apgar score at 5 min (lowest score if twins); median (IQR**)	8.5 (7–10)
Missing	6
**Infant ventilated at randomisation (either infant if twins); n (%**)	47 (35.6)

Data was provided by 103 participants at day 4 after birth and 91 participants at both days 14 and 21. Milk yield increased over time from a median of 155 g (IQR 66–296) on day 4 to 490 g (IQR 248–830) on day 21. Median expressing frequency was 5 (IQR 4–7) on day 4, and 6 (IQR 5–7) on days 14 and 21. There were minimal breast feeding attempts on any of these days. At 36 weeks’ PMA (when infants were on average 8.2 weeks of age), 64.9% of participants (74/114) were giving exclusive MOM and 86.0% were giving any MOM (98/114).

The median time to first expression attempt after birth was 6 hours (IQR 3–12), with strong left skew (online supplemental figure 1). Overall, 20.8% of participants expressed within 2 hours of birth (25/120), 30.8% expressed between 2 and 6 hours of birth (37/120) and 48.3% expressed more than 6 hours after birth (58/120). Time to first expression was missing for 12 participants.

10.1136/fetalneonatal-2023-326784.supp1 Supplementary data

### Association between time to first expression and milk yield

**Figure 1:**
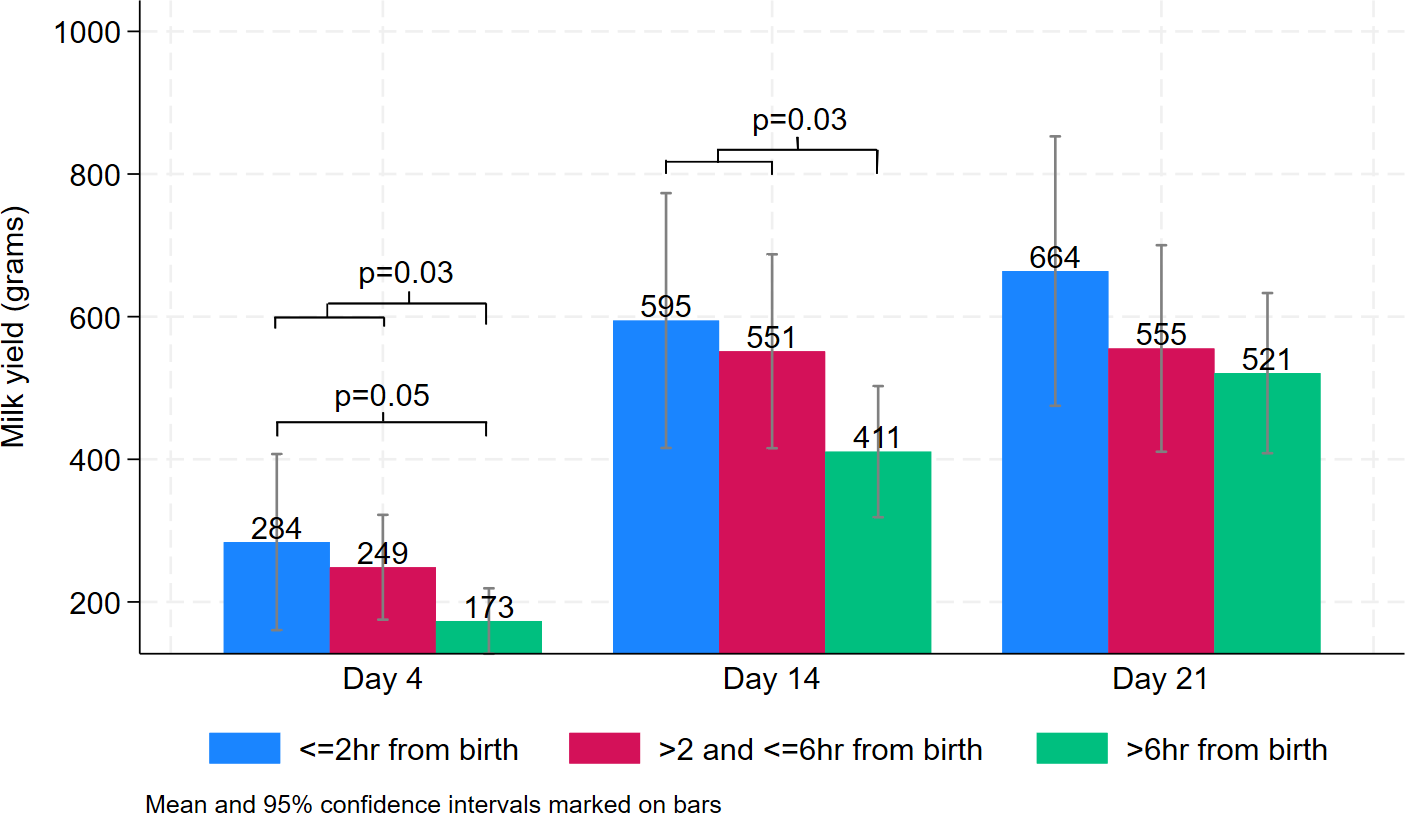
Expressed milk yield on days 4, 14 and 21 by time to first expression after birth.

Milk yield is shown in relation to two clinical recommendations; expressing within 2 and 6 hours of birth (figure 1).

A variety of ways to classify the time to first expression after birth in relation to milk yield were compared. At days 4 and 14, the association was strongest when the time to first expression was categorised as within 6 hours versus more than 6 hours after birth. When using a three-category variable, expressing within 2 hours of birth was associated with greater milk yield at day four than expressing more than 6 hours after birth, but no greater milk yield than expressing between 2 and 6 hours after birth. At day 21 there was no association between time to first expression and milk yield (table 2). The raw data for milk yield and expressing frequency are also shown using this 6-hour division in table 3.

**Table 2:** Milk yield according to adherence to international recommendations on time to first expression (unadjusted analysis)

	Day 4 milk yield (g)		Day 14 milk yield (g)		Day 21 milk yield (g)	
	Coefficient (95% CI)	P value	Coefficient (95% CI)	P value	Coefficient (95% CI)	P value
**Time to first expression attempt after birth (presented in four different formats):**						
**Per hour later**	−1.3 (−4.2 to 1.7)	0.40	−5.6 (−11.8 to 0.6)	0.07	−1.0 (−7.3 to 5.2)	0.75
**≤6 hours compared with >6 hours (international recommendation**)	88.3 (7.1 to 169.4)	0.03	155.7 (12.2 to 299.3)	0.03	73.6 (−91.4 to 238.7)	0.38
**≤2 hours compared with >2 hours (UK recommendation**)	79.6 (−24.6 to 183.7)	0.13	126.0 (−63.9 to 315.9)	0.19	127.5 (−75.5 to 330.5)	0.22
**Compared with ≤2 hours:**						
2 to ≤6 hours	−35.3 (−151.8 to 81.3)	0.55	−43.1 (−253.7 to 167.5)	0.69	−108.5 (−336.3 to 119.3)	0.35
>6 hours	−110.7 (−220.7 to −0.6)	0.05	−183.8 (−383.0 to 15.4)	0.07	−143.1 (−363.4 to 77.2)	0.20

.

**Table 3:** Lactation outcomes by time to first expression

	First expression attempt ≤6 hours after birth	First expression attempt >6 hours after birth
**Day 4 (N**)	52	47
**MOM quantity (g**)		
*Mean (SD*)	261.5 (235.6)	173.2 (159.8)
*Median (IQR*)	225 (67.9–362.7)	128.7 (66.2–238.5)
**Expressing frequency**		
*Mean (SD*)	4.9 (2.4)	5.1 (2.0)
*Median (IQR*)	5 (4–7)	5 (4–7)
**Day 14 (N**)	46	43
**MOM quantity (g**)		
*Mean (SD*)	566.4 (369.6)	410.7 (306.3)
*Median (IQR*)	543.3 (308.1–731.1)	321.7 (201.3–624.8)
**Expressing frequency**		
*Mean (SD*)	6.1 (1.8)	5.9 (2.1)
*Median (IQR*)	6 (5–7)	6 (5–7)
**Day 21 (N**)	50	39
**MOM quantity (g**)		
*Mean (SD*)	594.5 (412.1)	520.8 (356.3)
*Median (IQR*)	525.3 (287.1–860)	473.8 (231.6–777.7)
**Expressing frequency**		
*Mean (SD*)	6.0 (2.2)	5.5 (2.1)
*Median (IQR*)	6.5 (5–7)	6 (4.5–7)
**36 weeks’ PMA (N**)	57	50
**Exclusive MOM (n, %**)	39 (68.4)	31 (62.0)
**Any MOM (n, %**)	50 (87.7)	42 (84.0)

MOM, mother’s own milk; PMA, postmenstrual age.

### Interaction of time to first expression and expressing frequency

On all three time points, there was a statistically significant interaction between expressing frequency and time to first expression, with increased expression frequency associated with higher yield only in those who expressed within 6 hours of birth (p<0.005). This fully adjusted model included the randomised intervention, expressing mode (electric pump vs any other mode), simultaneous expression (vs sequential or single) and prior human milk feeding experience. No other baseline or expressing-related variables were retained within the model.

**Figure 2:**
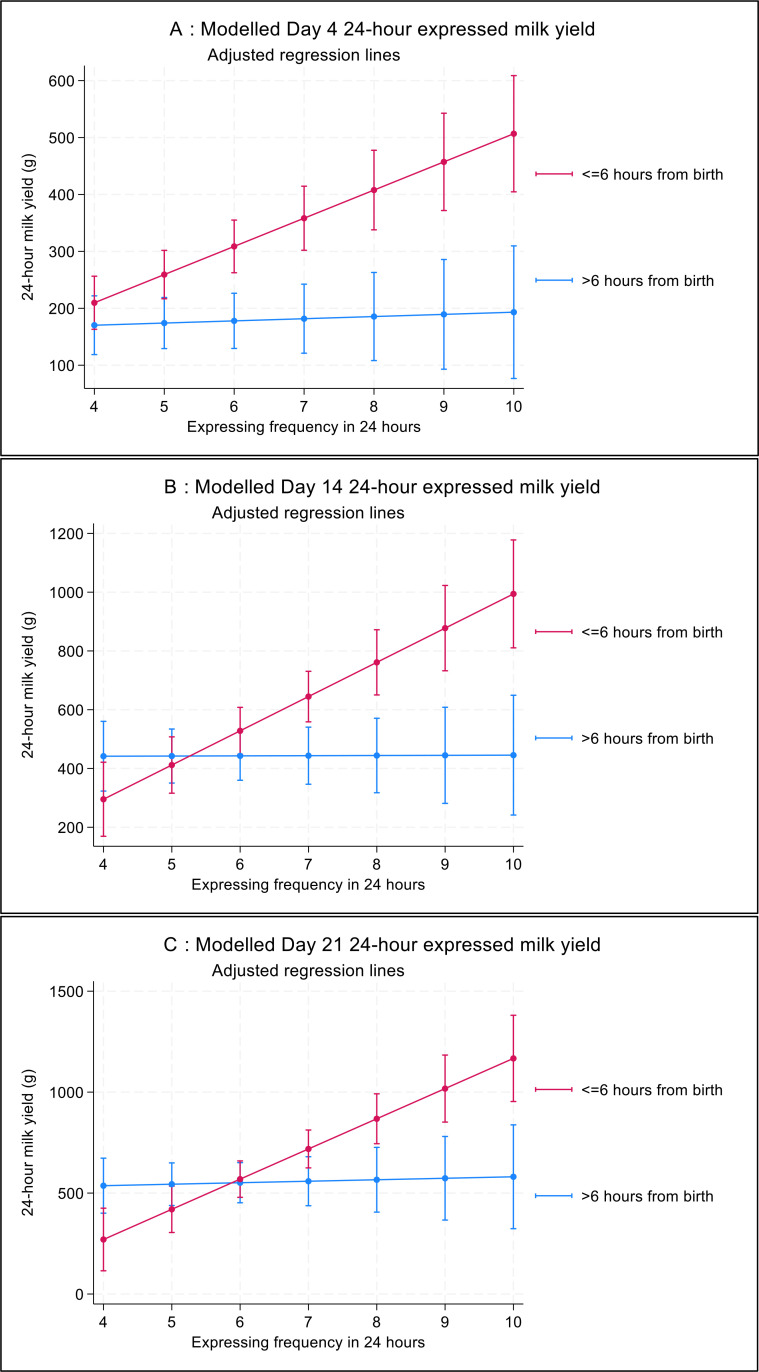
Adjusted expressed milk yield on days 4, 14 and 21, modelling an interaction of expressing frequency and time to first expression after birth. Adjustment is for randomised intervention, expressing mode (electric pump vs any other mode), simultaneous expression (vs sequential or single) and prior human milk feeding experience. The key refers to time to first expression attempt after birth.

There was a significantly higher milk yield for those expressing within 6 hours of birth when expressing at a frequency of five or more times on day 4, and seven or more times on days 14 and 21. This is demonstrated graphically in figure 2 (adjusted data). Online supplemental figure 2 shows the variability in milk yield in the raw data.

### Association between time to first expression and milk feeding outcome

There was no significant difference in exclusive MOM (relative risk (RR) 1.1, 95% CI 0.8 to 1.5) or any MOM (RR 1.0, 95% CI 0.9 to 1.2) at 36 weeks’ PMA for those expressing within the first 6 hours after birth compared with those expressing more than 6 hours after birth.

### Which participants expressed early?

Adjusted analysis showed that expressing within 6 hours of birth was more likely for those who had given birth vaginally (adjusted relative risk; aRR 1.5, 95% CI 1.1 to 2.1), those who were multiparous (aRR 1.6, 95% CI 1.1 to 2.2) and those living in less deprived areas (aRR per Index of Multiple Deprivation quintile of 1.2, 95% CI 1.0 to 1.4).

## Discussion

This study shows that expressing in the first 6 hours after very preterm birth was associated with a significant increase in milk yield in the weeks after birth, and also that those initiating expressing early gained a greater milk yield per extra expressing session. This suggests that they could potentially express less frequently. This information would be extremely motivating for parents, as families often find frequent milk expression burdensome and stressful at this time.^24–27^

Impacts were more clinically significant at day 4 than day 14 or 21, as at the later time points milk yield was only significantly increased for those expressing more than six times per day. By 36 weeks’ PMA no benefit could be seen for early expression in relation to any or exclusive provision of MOM. This weakening of impact over time suggests that later compensation is possible when mothers experience delayed first expression.

The final important insight gained from this study is that no further benefit of extremely early expression (within the first 2 hours of birth) was identified. Although the study did not have sufficient statistical power to detect a small increased benefit, these results suggest that it is unlikely that expressing within the first 2 hours after birth is associated with a further increase in milk yield that parents would find clinically significant, compared with expressing within the first 6 hours. This is important for clinical teams setting targets for early expression support, as expressing within the first 2 hours of birth can be extremely challenging for some families, particularly those experiencing postnatal complications or trauma reactions related to very preterm birth. Expressing within 6 hours is seen as more achievable by midwives.^9^

In considering the application of this evidence to clinical practice, it is useful to note that participants with caesarean birth, those who were primiparous and those who live in more deprived areas were all less likely to initiate expression early. These families should receive particular lactation counselling and support in preparation for very preterm birth and in the hours after birth.

### Setting the findings in context

This study had a shorter time to first expression than most previous cohorts (median of 6 hours in this study compared with 8,^28^ 9^16 19^ and 20–24 hours^29 30^ in other studies). Overall, the milk quantities and expressing frequencies noted in this cohort are comparable to many other studies.^19 29 31 32^ The rate of any and exclusive MOM was relatively high for the UK,^33^ which may be explained by the Southern English location of most of the recruiting units and the high motivation to exclusively MOM feed at baseline.

This study has replicated the previously unique finding of a statistical interaction between early first expression and ongoing frequency of expressing,^21^ although with different clinical impact. The prior study showed a greater difference in milk quantity between groups when expressions were infrequent, whereas we saw a greater difference when expressions were frequent. However, in the previous study, ‘early’ expression was defined as within 48 hours of birth, and the average time to first expression was over 55 hours. Our findings are therefore more relevant to modern practice and current recommendations for milk expression.

Our findings are concordant with the randomised trial^13^ report that expressing in the first 3 hours after birth is not superior to between 3 and 6 hours after birth. They are also consistent with other observational studies showing improved lactation outcomes when first expression takes place within 6 hours of birth,^15–17 19^ and those suggesting that the association weakens over time.^16 17 19 28^ A possible mechanism for this pattern is that early first expression may cause earlier secretory activation (lactogenesis II or milk ‘coming in’). This could explain the weakening association over time as it is possible for those with delayed secretory activation to ‘catch up’ to some extent.^34^ In addition, after secretory activation, new local and central factors come into play to influence milk quantity, which may affect the link between stimulation frequency and milk yield.^35^ Some randomised studies have reported earlier secretory activation with early expression.^14 36^

### Strengths and limitations

Key strengths of the study are the multicentre design and the broad population of included participants. This includes a wide range of sociodemographic variables and also a large number of mothers who experienced extremely preterm birth. As lactation outcomes tend to be worse after extremely preterm birth,^37 38^ as well as for those who are less educated, younger and from more deprived areas,^37 39^ this increases the external validity of the results.

Key limitations are the relatively small size of the study, its observational nature and the dependence on participant reports. For example, the peaks in the reported first expression at 12, 24, 48 and 96 hours suggest a level of estimation by participants, which may have reduced data accuracy. The analysis also lacked data on several potential confounders such as assisted reproduction^40^ and obesity,^40^ which could lead to residual confounding.

## Conclusions

This study has provided important information that clinicians can use to counsel and support families, and to motivate staff within quality improvement projects to reduce the time to first expression attempt after very preterm birth. The potential for early expression to reduce the burden of expressing frequency in the weeks after birth is novel and deserves further research.

## Data Availability

Data are available upon reasonable request. A review of all requests for sharing of the study data will take place as described in National Perinatal Epidemiology Unit Standard Operating Procedures on data sharing. If agreed, any sharing of the data collected during the study must be in accordance to the Nuffield Department of Population Health and University of Oxford policies.
