# Supplementary material for: Does extremely early expression of colostrum after very preterm birth improve mother’s own milk quantity? A cohort study
Source: Arch Dis Child Fetal Neonatal Ed. 2024 Mar 4;109(5):475–80. doi: 10.1136/archdischild-2023-326784 (PMC11347236; doi:10.1136/archdischild-2023-326784)

Supplementary Figures

Supplementary Figure 1: Histogram of time to first expression after birth, in hours

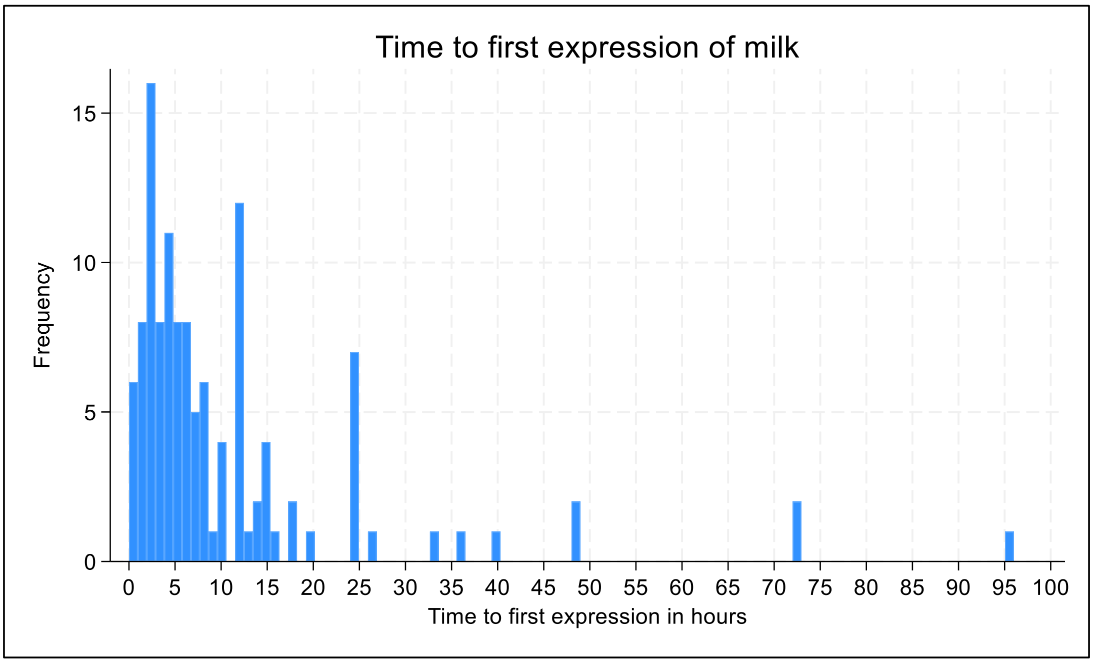

BMJ Publishing Group Limited (BMJ) disclaims all liability and responsibility arising from any reliance
placed on this supplemental material which has been supplied by the author(s)

Supplementary Figure 2: Daily expressed milk yield at day 4, 14 and 21 by expressing frequency and time to first expression after birth (raw data and unadjusted linear regression lines)

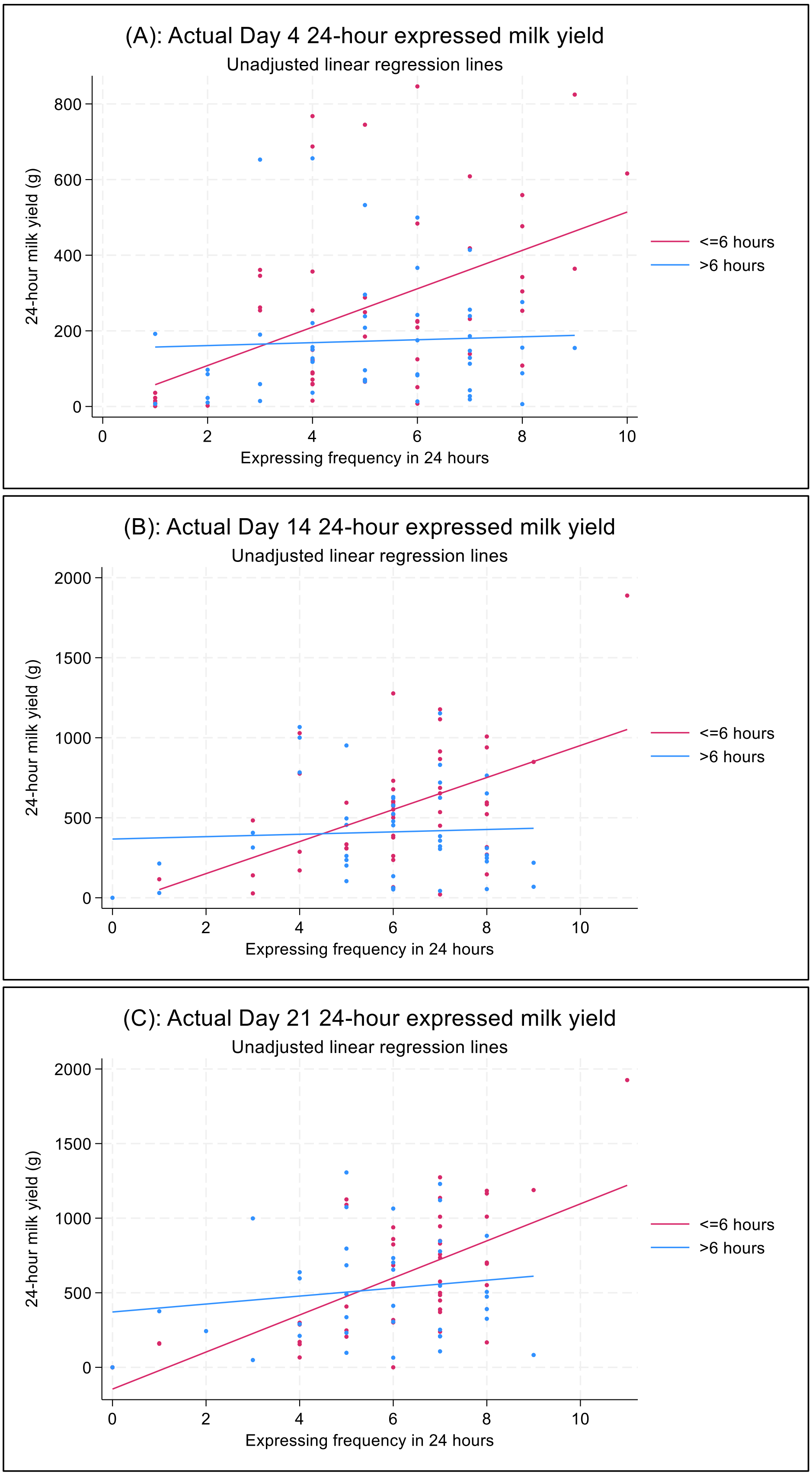

Supplement: Supplementary data [file fetalneonatal-2023-326784supp001.pdf]
